# New Crystal Form of Human Neuropilin-1 b1 Fragment with Six Electrostatic Mutations Complexed with KDKPPR Peptide Ligand

**DOI:** 10.3390/molecules28145603

**Published:** 2023-07-24

**Authors:** Ibrahima Goudiaby, Thérèse E. Malliavin, Eva Mocchetti, Sandrine Mathiot, Samir Acherar, Céline Frochot, Muriel Barberi-Heyob, Benoît Guillot, Frédérique Favier, Claude Didierjean, Christian Jelsch

**Affiliations:** 1 Université de Lorraine, CNRS, CRM2, F-54000 Nancy, France; i.goudiaby1592@zig.univ.sn (I.G.); eva.mocchetti@univ-lorraine.fr (E.M.); benoit.guillot@univ-lorraine.fr (B.G.);; 2 Université Assane Seck de Ziguinchor, Laboratoire de Chimie et de Physique des Matériaux (LCPM), 523 Ziguinchor, Senegal; 3 Université de Lorraine, CNRS, LPCT, F-54000 Nancy, France; therese.malliavin@univ-lorraine.fr; 4 Université de Lorraine, CNRS, LCPM, F-54000 Nancy, France; 5 Université de Lorraine, CNRS, LRGP, F-54000 Nancy, France; 6 Université de Lorraine, CNRS, CRAN, F-54000 Nancy, France; muriel.barberi@univ-lorraine.fr

**Keywords:** Neuropilin 1, variant, ligand, X-ray crystallography, molecular dynamics simulation, Hirshfeld interface, electrostatic influence

## Abstract

Neuropilin 1 (NRP1), a cell-surface co-receptor of a number of growth factors and other signaling molecules, has long been the focus of attention due to its association with the development and the progression of several types of cancer. For example, the KDKPPR peptide has recently been combined with a photosensitizer and a contrast agent to bind NRP1 for the detection and treatment by photodynamic therapy of glioblastoma, an aggressive brain cancer. The main therapeutic target is a pocket of the fragment b1 of NRP1 (NRP1-b1), in which vascular endothelial growth factors (VEGFs) bind. In the crystal packing of native human NRP1-b1, the VEGF-binding site is obstructed by a crystallographic symmetry neighbor protein, which prevents the binding of ligands. Six charged amino acids located at the protein surface were mutated to allow the protein to form a new crystal packing. The structure of the mutated fragment b1 complexed with the KDKPPR peptide was determined by X-ray crystallography. The variant crystallized in a new crystal form with the VEGF-binding cleft exposed to the solvent and, as expected, filled by the C-terminal moiety of the peptide. The atomic interactions were analyzed using new approaches based on a multipolar electron density model. Among other things, these methods indicated the role played by Asp320 and Glu348 in the electrostatic steering of the ligand in its binding site. Molecular dynamics simulations were carried out to further analyze the peptide binding and motion of the wild-type and mutant proteins. The simulations revealed that specific loops interacting with the peptide exhibited mobility in both the unbound and bound forms.

## 1. Introduction

Neuropilins (NRP1 and NRP2) are type I single-pass transmembrane glycoproteins expressed in all vertebrates and have many physiological roles. They act as co-receptors of a range of growth factors and other signaling molecules. A recent cryo-electron microscopy structure highlights the role of NRP1 in a ternary complex with a semaphorin protein and a plexin receptor, which altogether mediates signaling in neuronal axon guidance and other processes [1]. NRP1 also forms a ternary complex with vascular endothelial growth factor A 165 (VEGF-A165) and the receptor VEGFR2 [2]. This complexation is associated with intracellular signaling, mitogenesis, cell migration and angiogenesis [3]. Research has shown that NRP1 plays a significant role in the development and progression of various cancer types [4] and also more recently in the infectivity of severe acute respiratory syndrome coronavirus 2 (SARS-CoV-2) [5]. In particular, this transmembrane receptor has been suggested as a molecular therapeutic target for glioblastoma, with overexpression mainly due to endothelial cells of angiogenic phenotype and associated pro-tumor macrophages, both of which are linked to an unfavorable prognosis [6]. A recent review outlines the various functions of NRP1 in the context of cancer treatments [7].

**Figure 1:**
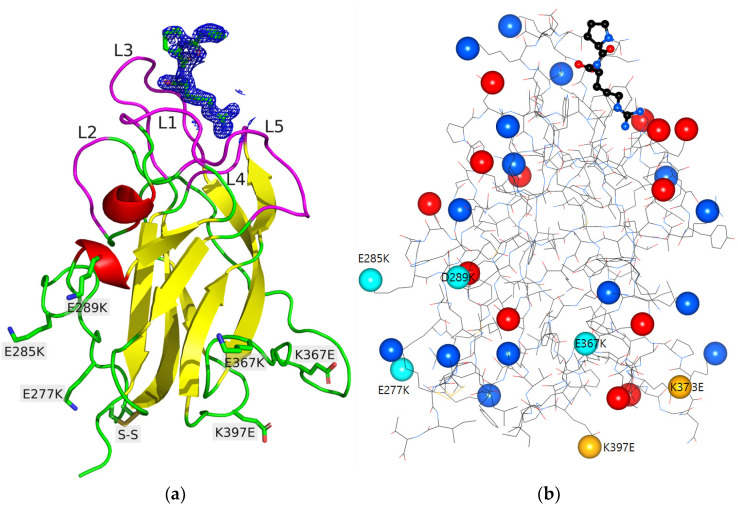
(**a**) Ribbon view of the crystal structure of NRP1-b1 hexavariant. The six mutations and the disulfide bridge Cys275-Cys424 are shown as sticks and labelled. The loops L1–L5 that line the VEGF-binding pocket are highlighted in magenta and labelled. The PPR moiety of KDKPPR peptide is shown as sticks with refined 2*mFo*-*DFc* electron density contoured at 1.0 σ. (**b**) Repartition of the charged residues in NRP1-b1 hexavariant. The positive (blue) and negative (red) charges are shown as spheres on the protein structure. The mutated residues with change in charge are labeled and are shown in light blue and light yellow. The PR residues of KDKPPR peptide are shown on the top of the figure.

The membrane protein NRP1 (as NRP2) contains a large N-terminal extracellular region (~850 amino acids (AA)), a single transmembrane domain (~25 AA) and a short C-terminal cytoplasmic domain (~45 AA) [8]. The ectodomain consists of five independent domains, where the first four (a1, a2, b1 and b2 domains) are involved in ligand binding, while the role of the last one (c domain) is still under debate (oligomerization, NRP1 homodimerization, etc.) [1]. The interdomain linkers are also important in the heterocomplex formation as spacers [1]. The determination of the crystal structure of the b1 domain provided the first structural insight at the atomic-level into NRP1 [9]. The interaction of NRP1 with VEGF has been extensively studied. Briefly, the NRP1-b1 domain folds into a distorted jelly roll barrel motif that is composed of two beta-sheets [9] (Figure 1a). The strands are connected by loops of varying length. The bottom of the beta-barrel core exhibits a triangular shape that contains an intramolecular disulfide bridge. At the top of the beta-barrel core, the loops are divided in six loop regions (L1–L6) to delimit the pocket of the positively charged tail of VEGF. The C-terminal arginine residue of VEGF-A165 is buried in this pocket and is a key feature of the binding. Numerous atomic experimental structures have been determined to characterize and/or inhibit the interaction of NRP1-b1 and VEGF using peptides or arginine derivatives as well as a fusion protein [10,11,12,13].

We have developed peptides combined with a photosensitizer to target NRP1 in the context of photodynamic therapy (PDT) to detect and treat glioblastoma [14,15,16]. Recently, a nanoparticle was designed that combines KDKPPR motif as a targeting peptide, porphyrin as photosensitizer and gadolinium chelate as contrast agent. This nanoparticle, called AGuIX@PS@KDKPPR, enables the detection of tumor tissue by magnetic resonance imaging and treatment by PDT [6,17,18]. The affinity of the nanoparticle for human NRP1 was validated, and it was found to be ten times lower than that of the free peptide (*K_D_* of 4.7 µM for AGuIX@PS@KDKPPR and *K_D_* of 0.5 µM for KDKPPR) [17].

In this study, the KDKPPR peptide was synthesized, and its molecular interactions with NRP1-b1 fragment were investigated by X-ray crystallography and molecular dynamics (MD) simulations. Previously, we have attempted to co-crystallize NRP1-b1 with a carbohydrate-based peptidomimetic [19]. However, we constantly obtained tetragonal crystals that were isomorphous to the crystals of the unbound protein. These crystals are unsuitable for obtaining structures of complexes because the site that binds the C-terminal tail of VEGF is obstructed by symmetry-related protein molecules [9]. We have used site-directed mutagenesis to modify the repartition of charges on the surface of NRP1-b1 to induce changes in the crystal packing. This approach is an efficient tool for crystallizing a protein in a new form and facilitating, for example, the formation of a protein–ligand complex in the crystal through co-crystallization or soaking techniques [20]. In this study, we successfully co-crystallized the KDKPPR peptide with a hexavariant of NRP1-b1 (Glu277Lys, Glu285Lys, Asp289Lys, Glu367Lys, Lys373Glu, Lys397Glu). An original crystal form was obtained where the VEGF-binding pocket is filled by the KDKPPR peptide and is located in large spaces connected by solvent channels in the crystal packing. The structure of the NRP1-b1/KDKPPR complex was analyzed by MD simulations and innovative tools based on a multipolar electron density model [21].

## 2. Results and Discussion

### 2.1. Design of the NRP1-b1 Hexavariant

**Figure 2:**
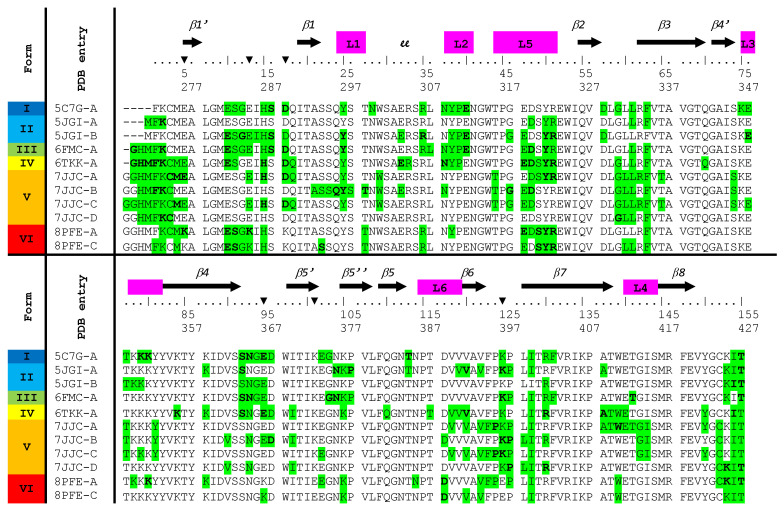
Highlights (in green) of the NRP1-b1 residues involved in contacts with a neighboring monomer in the crystal forms I to VI. Form VI corresponds to the NRP1-b1 hexavariant. Contacts are defined as residues with a proximity of less than 4 Å. Residues in bold characters forms intermolecular hydrogen bonds. The positions of the mutations have been highlighted with triangles above the two sets of residue numbering.

The charge distribution on the surface of NRP1-b1 was significantly altered in order to promote the formation of a new crystal form, based on a visual inspection of the model. The point mutations were chosen to be far away from the VEGF-binding pocket to minimize disruption of the peptide-binding site (Figure 1b). Specifically, six charged residues on the protein surface were mutated to residues of opposite charge: Glu277Lys, Glu285Lys, Asp289Lys, Glu367Lys, Lys373Glu and Lys397Glu. Mutation of these residues did not lead to isoelectric conservation. In fact, the estimated isoelectric point of the hexavariant was 1 unit higher than that of the native protein, with a value of 9.2 for the hexavariant and 8.0 for the wild type. These choices resulted in the creation of a highly electropositive region around the Glu285Lys, Asp289Lys and Glu277Lys mutations, consisting of seven positively charged residues (Lys274, Lys277, Lys285, Lys289, Arg334, Arg402 and Lys425). Three of them (Lys277, Lys285 and Lys425) form hydrogen bonds with symmetry-related molecules in the crystal of the hexavariant (see below, Figure 2 and Table S1).

### 2.2. Crystal Structure of the NRP1-b1 Hexavariant

#### 2.2.1. Description of the Structure

The hexavariant (Glu277Lys, Glu285Lys, Asp289Lys, Glu367Lys, Lys373Glu, Lys397Glu) of the NRP1-b1 domain crystallized in the *P*3_2_21 space group with a novel packing arrangement. The asymmetric unit contained two chains (A and C), each with a KDKPPR peptide (chains B and D) in its VEGF-binding site, plus 377 water molecules and an acetate ion. Only the last three residues of the peptide (PPR) in both monomers were included in the refined structure (Figure 1a). Positive residual peaks in the difference electron density maps persisted around the first proline residue of the KDKPPR peptide. These peaks were slightly stronger in monomer D. We made several attempts to improve the final 2*mFo*-*DFc* electron density map, such as modeling an additional residue in the peptide or modeling alternative conformations for the first proline residue. However, no satisfactory model has emerged from any of these efforts. The two monomers were nearly identical, with an overall coordinate root mean square deviation (RMSD, Å) of 0.30 Å on the 154 Cα atoms common to both chains. Slight conformational differences were observed at the very first and last residues of the two monomers, due to their different involvement in packing contacts. This probably also explained the differences observed at the neighboring disulfide bridge Cys275-Cys424 of the two monomers. Indeed, in monomer A, it showed two alternative conformations, one of which was rather ill-defined, whereas only one conformation was observed in monomer C. The same argument concerning packing effects probably applied for the slight differences observed in some side chain conformations (Phe335) and sometimes in some main chain regions (Glu374-Pro378, Pro396-Pro398). The mutations did not affect the protein fold, since the current hexavariant model showed an overall RMSD of 0.51 Å with the wild-type structure (PDB entry 1KEX, [9]), compared to a 0.30 Å RMSD between the hexavariant independent monomers (A and C). Four of the mutations (Glu277Lys, Glu285Lys, Asp289Lys and Lys397Glu) resulted in no apparent change in the main chain fold. On the contrary, Glu367Lys and Lys373Glu were located in regions with larger observed displacements: the Cα atom of residue 367 underwent a 1.75 Å shift due to an overall movement of region Ser363-Trp369, while the *ψ* angle of Gly375 rotated 180° in the rearrangement of the Glu373-Pro378 loop. Both of these observations are most likely the consequence of the change in crystal packing and protein–protein contacts (Figure 2), rather than the direct influence of the mutations on the polypeptide conformation.

#### 2.2.2. Crystal Packing and Intermolecular Contacts

The mutations induced a new crystal packing in which a few introduced residues (Lys277 and Lys285 in chain A) were involved in modified intermolecular interactions. The two independent hexavariant models (chains A and C) have similar molecular environments. Indeed, more than half of the contacts are identical in both chains (Table S1). The KDKPPR peptide is not involved in the crystal cohesion. The hexavariant crystal contains large solvent spaces (volume of approximatively 30,000 Å^3^, Figure S1) into which it seems possible for small molecules to diffuse because they are connected by solvent channels. The N-terminal parts of the two independent KDKPPR peptides (KDK moiety, chains B and D) are located in the bulk solvent, while their C-terminal parts are tightly bound in the pocket that would welcome the C-terminal tail of VEGF, in chains A and C, respectively (see below). The electron density for the N-terminal regions of chains B and D was too weak to build a model, probably because these regions exhibited dynamic disorder. 

We compared the hexavariant crystal form with those of the NRP1-b1 domain available in the Protein Data Bank [22]. Five structures of NRP1-b1 with distinct unit cell parameters were found (Figure 2, Table S2). The asymmetric units contain one to four independent chains. The crystal structure of the tetragonal form I represents the unbound state of NRP1-b1, in which the VEGF-binding site is obstructed by symmetry-related molecules. All other crystal forms were obtained by co-crystallization of NRP1-b1 with arginine or close derivatives (crystal forms II to V). The intermolecular environment analysis revealed that crystal forms II and III have similar packings (*P*2_1_ space group with two independent chains and *P*4_1_ space group with one independent chain, respectively) (Figure S2). We also noticed that in all cases, at least one independent ligand was positioned at the interface between two adjacent NRP1-b1 monomers (Figure S3). Their presence was probably necessary for the cohesion of these crystal packings. That is why we worked with a variant, trying to find a new crystal packing where the ligand only contacts the protein to which it is bound.

### 2.3. Protein Ligand Interaction

#### 2.3.1. Description of Ligand Binding

**Figure 3:**
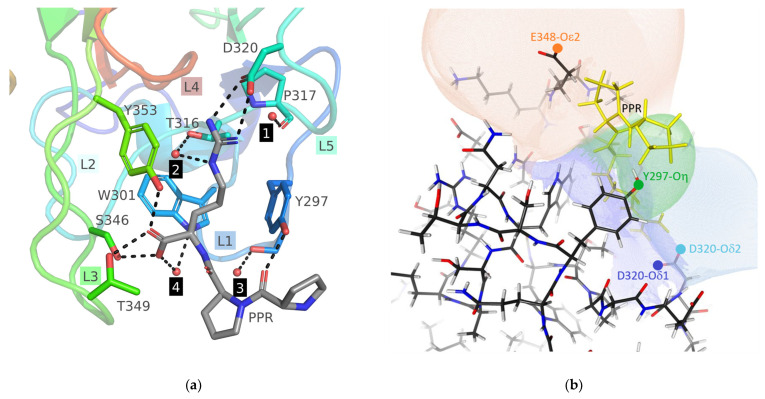
(**a**) Structure of the binding site of NRP1-b1 hexavariant in complex with the KDKPPR peptide. The pocket is mainly composed of five loops (L1 to L5), which are respectively colored blue, cyan, green, red and turquoise. The crystallographic model of the peptide includes only the PPR moiety, represented as sticks. The NRP1 residues in the close proximity of the peptide are also depicted as sticks. The four structural water molecules are highlighted as spheres, while hydrogen bonds are illustrated as dashed sticks. Various labels are provided to enhance clarity, indicating loops, peptide, residues and water molecules. (**b**) Nucleophilic Influence Zones associated with the oxygen atoms Asp320-Oδ1 (dark blue), Asp320-Oε2 (light blue), Tyr297-Oη (green) and Glu348-Oε2 (major conformer, orange) in the vicinity of the ligand-binding site of monomer A. The corresponding atomic nucleophilic sites are indicated by colored circles, and the PPR moiety of the KDKPPR peptide is highlighted in yellow.

The KDKPPR peptide was bound in the VEGF-binding pocket formed by loops L1 to L5 of NRP1-b1, with its C-terminal arginine positioned in a very similar manner to what was described in detail by [11]. Briefly, its guanidine group forms a salt bridge involving two hydrogen bonds with Asp320 (L5), while the aliphatic part of its side chain is stacked between the phenyl rings of Tyr297 (L1) and Tyr353 (L3) (Figure 3a). The arginine residue of the ligand is also tightly bound at the main chain by residues of L3, with one of its carboxylate oxygen atoms hydrogen-bonded to the side chain hydroxyl groups of Thr349 and Tyr353 and the second one to the lateral chain of Ser346. The last strong anchoring of the KDKPPR peptide arises from the main chain carbonyl group of the first proline, which forms a hydrogen bond with the hydroxyl group of Tyr297 (L1). The first three residues of the peptide apparently found no preferred binding to NRP1-b1 that could have fixed them in a given conformation and made them visible in the electron density.

Ordered water molecules were checked and found identical to those discussed by [11]. Four structural water molecules in the peptide-binding site (HOH A690, A666, A717 and B101 for chain A and HOH C570, C549, C596 and D101 for chain B) were included in calculation of the contacts enrichment ratio (see below, Figure 3a). 

#### 2.3.2. Electrostatic Influence of the Protein on the Peptide

The VEGF-binding site of NRP1-b1 is occupied by the PPR moiety of the KDKPPR peptide, with its electrophilic C-terminal arginine residue forming a salt bridge with Asp320. For this reason, it is interesting to study the electrostatic influences from the whole NRP1 protein on the peptide from the point of view of Nucleophilic Influence Zones (NIZ) [23] (Figure 3b). A NIZ represents the volume containing all the electric field lines converging to a specific nucleophilic site, often an oxygen atom in proteins. Therefore, an electrophilic ligand within this space, like a positive charged entity, experiences attractive electrostatic forces directed toward the corresponding nucleophilic site. NIZs associated with the relevant oxygen atoms involved in the binding of the PPR moiety were calculated excluding ligand atoms and using CHARGER program [24].

As anticipated, the NH_2_ groups of the arginine guanidinium of the KDKPPR peptide are influenced by their respective hydrogen-bonded oxygen atoms of Asp320 (dark blue surface for Oδ1 atom and light blue surface for Oδ2 atom) (Figure 3b). The NIZ of the Glu348-Oε2 atom (orange surface) covers the position of one peptide proline residue, while the NIZ of the Tyr297-Oη atom (green surface) encompasses the majority of the other peptide proline residue. Hence, these NIZs illustrate the forces acting on both proline residues, specifically exerting an attraction on their hydrogen atoms, thus stabilizing the ligand conformation by pulling one proline residue towards Glu348 and the other towards Tyr297. 

Given the close proximity of the solvent dielectric medium and the distances between the proline residues involved and the generators of the discussed NIZs (i.e., Glu348-Oε2 and Tyr297-Oη), it is likely that the electrostatic stabilization is relatively weak but cannot be disregarded. Electrostatic forces, being long-range interactions, play a role, and the presence of numerous charged hydrogen atoms in the pyrrolidine rings of prolines facilitates favorable interactions with the negatively charged oxygen atoms of the side chains of Glu348 and Tyr297, supporting our interpretation.

However, there are other factors contributing to the stabilization of the KDKPPR peptide that deserve attention. One such factor is the strong hydrogen bond between the hydroxyl group of Tyr297 and the carbonyl oxygen atom of the first proline residue in KDKPPR (Figure 3a). Furthermore, a stabilizing van der Waals contact can be inferred from the proximity of the Glu348-Cγ hydrogen atoms and the Cγ hydrogen atoms in the second proline residue of the KDKPPR peptide.

Furthermore, since electrostatic influences participate in the driving of ligand diffusion across the solvent toward a protein-binding site [25], the NIZs also provide insights on the electrostatic forces originating from the protein residues and directing the ligand during its approach. Here, the NIZs of Glu348-Oε2 and Asp320-Oδ2 atoms extend beyond the protein surface, suggesting their potential contribution to the electrostatic steering effect that facilitates electropositive ligand fixation in the VEGF-binding site of NRP1-b1 (Figure 3b).

#### 2.3.3. Hirshfeld Surface and Contacts Enrichment Ratio

The Hirshfeld surface [26] between the peptide and the protein was calculated with MoProViewer software [27]. The Hirshfeld surface allows the analysis and visualization of intermolecular interactions. The contact enrichment ratio *E*_XY_ between chemical species X and Y is obtained by comparing the actual *C*_XY_ contacts with those calculated as if all contact types had the same probability of forming [28]. The equiprobable proportions *R*_XY_ are derived by probability products from the chemical proportions on the Hirshfeld surface. An *E*_XY_ enrichment ratio greater than unity for a particular contact between chemical species X…Y indicates that these are over-represented. The chemical nature of the contacts and their enrichment in the complex of NRP1-b1 with KDKPPR peptide are shown in Table 1. The proportions of contact types are very similar in the two independent monomers of NRP1-b1 (correlation of *C*_XY_ contact type proportions of 99.9%).

The less polar Hc hydrogen atoms bonded to carbon were distinguished from the more electropositive Ho/n atoms bound to oxygen or nitrogen. Four structural water molecules in monomer A (and their equivalent in monomer B, see above) in the binding cleft were kept and attributed to the protein in the complex. Obviously, the O…Ho/n hydrogen bonds are strongly attractive from an electrostatic point of view and are overrepresented (*E* = 2.58, Table 1). Representing 17.4% of the interaction surface, they are recognized as the most favored contacts. This concerns interactions between C=O and COO^−^ acceptors and N-H, NH_2_ and O-H hydrogen bond donors. The O…Hc weak hydrogen bond contacts represent 15.3% of the interaction surface and can be considered as weakly favored contacts, with an enrichment ratio of *E* = 1.20. Some enrichment ratios close to zero concern the O…O and Hn/o…Hn/o contacts, which are absolutely avoided in the protein/ligand complex because they concern repulsive self-contacts between charged species.

Occupying the largest contact area (21.3%), non-polar Hc…C contacts are significantly over-represented (*E* = 1.80) and consist in particular of C-H…π interactions involving the aromatic rings of Tyr239, Tyr183 and Tyr187. The Hc…Hc contacts represent 10.3% of the surface and can be considered as weakly disfavored contacts, as they present an enrichment ratio lower than unity (*E* = 0.81). The Ho/n…W (oxygen atoms of water molecules) contacts involving the four structural water molecules represent 6.9% of the interaction surface and can be considered as significantly favored contacts, with *E* = 1.88. The four water molecules interact essentially with Ho/n and secondarily with Hc atoms.

The hydrophobic and hydrophilic atoms were regrouped in order to analyze the interactions between the two subgroups. The N contact surface occurs on sp^2^ peptide and guanidinium nitrogen atoms (N without electron lone pair) and was considered hydrophobic together with the C and Hc atoms. The peptide and protein interaction surfaces are constituted by more hydrophobic (57.1 and 52.6%, respectively) than hydrophilic atoms. The protein/ligand complex shows an enrichment of contacts between hydrophilic atoms (*E* = 1.26) and between hydrophobic atoms (*E* = 1.38). On the other hand, despite the mild enrichment of the weak hydrogen bonds of O…Hc, the cross contacts Hphob x Hphil are strongly under-represented (*E* = 0.69), which indicates a good partitioning of hydrophilic and hydrophobic contacts. 

In summary, the protein/ligand complex is mainly maintained by over-represented strong O…Ho/n interactions, which correspond notably to the salt bridge anchoring Asp206 and the arginine residue of the peptide. Secondarily, more moderately enriched interactions also play an important role, such as weak C-H…O hydrogen bonds and hydrophobic contacts, notably between Hc and C atoms. The enrichment values agree with trends found in studies of interactions in several families of oxygenated and nitrogenated hydrocarbon molecules [28,29]. The strong hydrogen bonds such as O…Ho/n are significantly enriched; in the case of small-molecule crystals, the over-representation reaches even larger values beyond 10. 

Concerning the weak C-H…O hydrogen bonds, they tend to occur in a moderately under-represented way in crystal structures of small molecules containing both strong H-bond donors and acceptors (such as alcohols for example), due to the competition of strong H-bonds. On the contrary, in the present protein/peptide interface, they appear slightly enriched. This can be explained by the excess of strong H-bond acceptors on the protein (*S*_O_ + *S*_W_ = 31.9%) compared to *S*_Ho/n_ = 22.9% of strong H-bond donors on the peptide.

### 2.4. Molecular Dynamics

**Figure 4:**
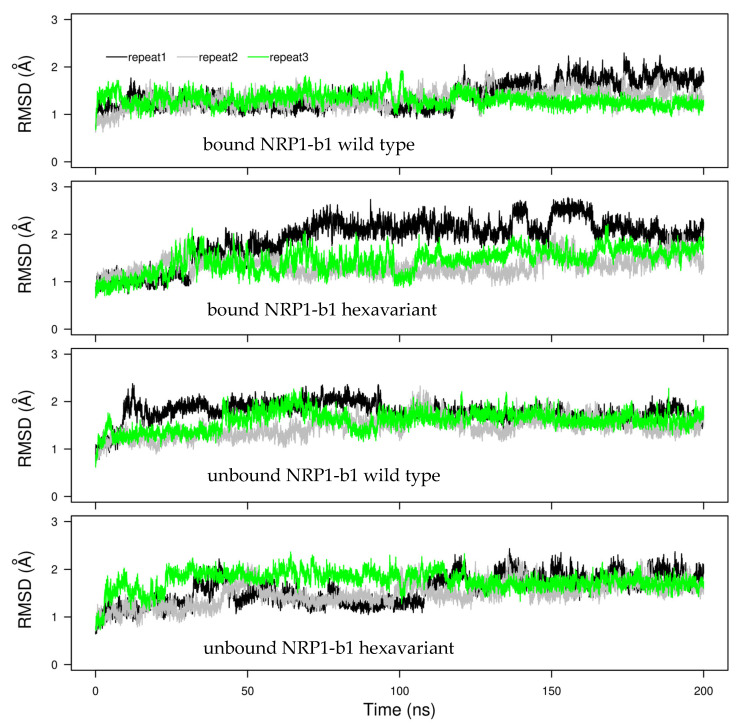
Coordinate RMSD (Å) calculated on the backbone heavy atoms of NRP1-b1 with respect to the initial X-ray crystallographic structure. The curves measured on the triplicated trajectories are colored in black, green and gray, respectively.

Coordinate RMSD of NRP1-b1 was monitored along the molecular dynamics (MD) trajectories and stabilized after 50 ns in a range of 1–2 Å. Similar coordinate drift was observed for the bound and unbound proteins, as well as for the mutated and wild-type sequences. Only one copy of the mutated protein in complex with the peptide displays slightly larger coordinate drift (Figure 4).

**Figure 5:**
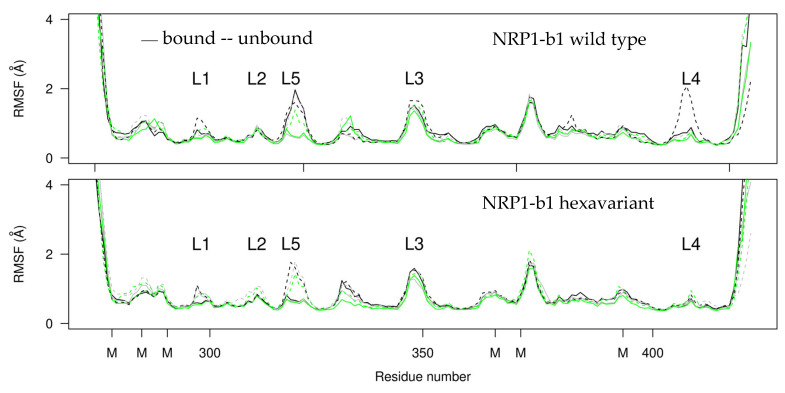
Atomic root mean square fluctuations (RMSFs, Å) calculated along the molecular dynamics (MD) trajectories by superimposing the heavy backbone atoms of NRP1-b1 on the corresponding atoms in the initial crystal structure. The upper panel corresponds to the trajectories recorded on the WT protein, while the lower panel corresponds to the trajectories recorded on the NRP1-b1 hexavariant used to determine the crystal structure. The curves measured on the triplicated trajectories are colored black, green and grey, respectively. They are plotted as solid and dashed lines for NRP1-b1 in complex with the peptide and for the unbound form, respectively. The positions in the sequence of the mutated residues are marked with the letter ‘M’. The NRP1 loops interacting with the peptide are labelled as defined in the text.

The atomic root mean square fluctuations (RMSFs, Å, Figure 5) of NRP1-b1 display quite superimposed profiles for all MD trajectories, with the unbound protein (dashed line) showing more mobile regions in particular for the loop of residues 317–322, which corresponds to the loop L5 of the VEGF-binding pocket. The very similar fluctuation profiles for the variant and WT forms show that the mutations introduced to alter crystal packing do not introduce a major bias in the dynamics of the protein. The N-terminal region of the KDKPPR peptide interacting with NRP1-b1 displays large fluctuations along the trajectories, in agreement with the invisible electronic density for this part of the peptide. 

The root mean square thermal displacements (RMSTDs) of the Cα atoms in the crystal structure were derived from the thermal parameters using the formula $\mathrm{RMSTD}=\sqrt{B/8\pi^{2}}$. This estimation is meaningful because we obtained a high-resolution structure (1.35 Å, Table 2) [30]. The values were then compared to the average RMSF obtained from three MD simulations of NRP1-b1 hexavariant in complex with the peptide (Figure S4). RMSTD and RMSF show similar profiles, indicating a general agreement between both indicators. However, the values of RMSF are noticeably larger (up to 1.7 Å) in the most dynamic regions of the polypeptide chain, while RMSTDs consistently remain below 0.75 Å. One plausible explanation for this discrepancy is that proteins in the crystalline state typically exhibit reduced mobility compared to their counterparts in solution. Overall, RMSF and RMSTD show a correlation coefficient of 0.735, indicating a moderate correlation between the two parameters. The values of RMSF are noticeably larger (up to 1.7 Å) in the most dynamic region of the polypeptide chain, while RMSTDs consistently remain below 0.75 Å. Several MD simulations of the literature [31,32,33,34] have also shown larger fluctuations in solution compared to the crystal environment. Our observations agree with these references. 

Looking more closely at the RMSF profiles (Figure 5), the loops interacting with the peptide all display fluctuation peaks whether the peptide is absent or present. The loop 347–350 (L3 loop) bearing Glu348 displays the same mobility. By contrast, a group of loops clustered on the other side of the binding side, the loop 296–300 (L1) bearing Tyr297 and Asn300, the loop 310–313 (L2) bearing Glu312 and the loop 318–322 (L5) bearing Glu319, are more mobile in the absence than in the presence of the ligand. Somehow, the two sides of the binding pocket behave differently with respect to the ligand. One moiety (L1, L2 and L5) stabilizes upon binding, while the other moiety (L3) retains the same mobility. A recent MD study by Alshawaf et al. [35] found similar RMSF profiles in NRP1-b1 when complexed with specialized metabolites 3-*O*-methylquercetin and esculetin. Specifically, the fluctuations in the esculetin/NRP1-b1 complex were similar to those observed in our study of the unbound form, while the 3-*O*-methylquercetin/NRP1-b1 complex was more similar to our complex with the peptide. Notably, the study found that 3-*O*-methylquercetin had a more favorable energy of interaction with NRP1-b1 than esculetin [35]. Our “bound” RMSF profile may be indicative to a state of NRP1-b1 that allows stable interactions with a ligand.

Comparing the fluctuation profiles of the WT and of the mutated sequences of NRP1-b1 (Figure 5), only one major difference can be noticed: the loop 411–416 (L4), interacting with the peptide, displays a fluctuation peak for one of the unbound trajectories on the WT sequence, whereas this loop is quite rigid in all trajectories recorded for the modified sequence.

The loops 282–289 and 373–378, located in the bottom of the structure, display high mobility in all conditions. The loop 282–289, containing the charged and polar sequence ESGEIHSD, becomes more mobile in the absence of peptide. The sequence ESGEIHSD (residues 282–289) is located at the surface of b1 domain close to the surface of a2 domain in NRP1 structures, which contain a2, b1 and b2 domains (PDB entry 2QQM, [36]) or a1, a2, b1 and b2 domains (PDB entry 4GZ9, [37]). A similar configuration is also visible in the more recent cryo-EM structure of the Sema3A/PlexinA4/NRP1 tripartite complex [1]. As the b1 and a2 interface do not form direct contact in any of these structures, it is difficult to speculate on the precise functional effect of the mobility of the region, but this mobility may have an influence on the propagation of conformational signals during the physiological processes.

## 3. Materials and Methods

### 3.1. Protein Production and Purification

The gene of NRP1-b1 hexavariant (residues Met272 to Thr427 of NRP1 with mutations Glu277Lys, Glu285Lys, Asp289Lys, Glu367Lys, Lys373Glu, Lys397Glu) was cloned into pET15b-NRP16mut-6His-3C (Novagen, Pretoria, South Africa) and expressed in *Escherichia coli* after induction at 18 °C. Cells were grown in Terrific-Broth at 18 °C to optical density (OD) = 0.36. After 8 h 25 min at 18 °C, they were induced with 0.2 mM Isopropyl β-D-1 thiogalactopyranoside. After growth at 18 °C for 10 h 25 min to OD = 0.7, cells were harvested by centrifugation, lysed and centrifuged. Proteins were purified on HIS-Select nickel affinity resin (Sigma-Aldrich, St. Louis, MO, USA) in 50 mM Tris (pH 8.0) and 300 mM NaCl with a 250 mM imidazole gradient. The protein was further purified by gel filtration using a Superdex75 HiLoad 16/60 column (GE Healthcare, Piscataway, NJ, USA) equilibrated and run in buffer A (50 mM Tris, pH 8, 300 mM NaCl, 20 mM imidazole). Analytical gel filtration experiments were performed using a Superdex 75 10/16 column (GE Healthcare) in buffer A. The protein was concentrated to 63 mg/mL by centrifugation on an Amicon ultrafiltration unit with a 10 kDa molecular weight cutoff, in a solution of Tris/HCl pH 8, NaCl 50 mM. 

### 3.2. KDKPPR Synthesis on Solid Phase

The KDKPPR peptide was synthesized using the automated ResPepXL peptide synthesizer, with a Fmoc/tBu methodology. The side chains of arginine, lysine and aspartic acid were protected by Pbf, OtBu and Boc groups. A Fmoc–Arg(Pbf)–Wang resin swelled in DCM was used. The Fmoc group was removed by a piperidine solution (20% in DMF), and this step was performed two times (the first for 4 min and the second for 7 min). Then, the next AA was grafted by adding an excess of Fmoc–AA–OH (6 eq), HBTU (5 eq), NMP (3 eq) and NMM (10 eq) in DMF, and this step was repeated two times for 18 min. A last step of capping, using a solution of acetic anhydride (5% in DMF), was performed for 5 min to trap all amino functions that did not react. Deprotection, coupling and capping steps were repeated until the end of the synthesis of the peptide. After a last Fmoc deprotection, the resin was dried under vacuum and then cleaved (with full deprotection of lateral chains) using TFA/TIPS/water (92.5/2.5/5, *v*/*v*/*v*) for 2 h. The acidic resin was filtered and washed with DCM and EtOH. The filtrate was dried under vacuum, and the compound was precipitated in diethylether by centrifugation. TSK gel Amide-80 column was used for HILIC purification of KDKPPR peptide using acetonitrile/water (0.1% TFA; 95/5 (*v*/*v*) to 55/45 (*v*/*v*) gradient) for 15 min, followed by an isocratic elution (0.1% TFA; 55/45, *v*/*v*) for 10 min at a flow of 12 mL/min (*R_t_* = 20.8 min). KDKPPR was isolated as a white powder with a yield of 64% and a purity of 95% (UV-vis detection at 214 nm).

### 3.3. Crystallization

Crystallization was conducted by sitting-drop vapor diffusion method. The reservoir solutions were prepared by mixing 0.3 µL of commercial reservoir solutions (screens) and 0.3 µL of protein solution. The crystallization trials of NRP1-b1 hexavariant were carried out in the presence of the KDKPPR hexapeptide at 20 °C. Crystals appeared with a JCSGplus solution composed of 0.2 M ammonium citrate, 0.1 M bis-tris pH 5.5 and 25% *w*/*v* PEG 3350.

### 3.4. X-ray Diffraction Data Collection and Crystal Structure Determination

Crystals of NRP1-b1 hexavariant that appeared suitable for X-ray diffraction data collection were quickly soaked in their mother liquor supplemented with 20% glycerol (*v*/*v*), before flash freezing in a nitrogen stream at 100 K. Preliminary X-ray diffraction experiments were carried out in-house on an Agilent SuperNova diffractometer (Oxford Diffraction, Oxford, UK) equipped with a CCD detector, and high-resolution data were further collected by ESRF synchrotron on beamline BM07 (Grenoble, France). The data set was indexed and integrated with XDS [38], scaled, and merged with Aimless [39] from the CCP4 suite [40]. The atomic structure was solved by molecular replacement using MOLREP [41] with the coordinates of NRP1-b1 wild type (PDB code 5C7G, [19]) as the search model. The structure was manually adjusted with Coot [42] and refined with Buster [43]. Structure validation was performed with MolProbity [44] and the wwPDB validation server (http://validate.wwpdb.org, (accessed on 22 december 2022)). Diffraction data and refinement statistics are shown in Table 2. Figures of the protein structures were generated with Pymol (Schrödinger LLC, NewYork, NY, USA), MoProViewer [27] and Ligplot+ [45], and cleft volume calculations were performed with 3V [46]. Coordinates and structure factors were deposited in the Protein Data Bank (PDB ID: 8PFE, DOI:10.2210/pdb8pfe/pdb).

### 3.5. Nucleophilic Influence Zones

The Nucleophilic Influence Zones were calculated from the electrostatic potential using Charger module of MoProviewer [24,27]. The electrostatic potential was generated from an electron density model, based on transferred multipolar parameters of the ELMAM2 library [21].

### 3.6. Hirshfeld Analysis

MoProViewer software [27] was used to investigate the intermolecular interactions and the contacts enrichment on the Hirshfeld interface between the protein molecules and the ligand peptides. The intermolecular interactions were evaluated by computing the enrichment ratios (Table 1) in order to highlight which contacts are favored. The enrichment values are obtained as the ratio between the proportions of actual contacts *C*_XY_ and the equiprobable (random) contacts *R*_xy_, the latter being obtained by probability products (*R*_XY_ = *S*_X_ *S*_Y_). 

Contacts X…Y, which are over-represented with respect to the share of X and Y chemical species on the Hirshfeld surface, have enrichments larger than unity. They are likely to represent interactions that are attractive from an electrostatic point of view and shall be the driving force in the complex formation [28]. Interactions between atoms that have electric charges of the same sign are repulsive and are generally under-represented (*E* < 1).

### 3.7. Molecular Dynamics

The protein and peptide chains C and D were selected from the X-ray crystallographic structure. The hydrogen atoms were added, and the flip of side chains was optimized using the Molprobity server [44]. The NRP1-b1 crystal structure with six mutations was unmodified, whereas for the wild-type (WT) system, the mutations Lys277Glu, Lys285Glu, Lys289Asp, Lys367Glu, Glu373Lys and Glu397Lys were introduced to return to the WT sequence of NRP1 for both protein sequences. The bound and unbound systems were simulated.

For each previously described system, the protein was embedded in a water box. Sodium and chloride counterions were added to obtain an ionic concentration of 0.15 M. The total number of atoms was about 31,000 in both cases. All MD simulations were performed using NAMD 2.14 [47], with the CHARMM36 force field [48] for protein and the TIP3P model for water [49]. A cutoff of 12 Å and a switching distance of 10 Å were used for non-bonded interactions, while long-range electrostatic interactions were calculated with the Particle Mesh Ewald (PME) method [50]. The RATTLE algorithm [51] was used to keep rigid all covalent bonds involving hydrogen atoms, enabling a time step of 2 fs. At the beginning of each trajectory, the system was minimized for 20,000 steps, and it was then heated up gradually from 0 K to 310 K in 31,000 integration steps. Finally, the system was equilibrated for 1 ns in the NPT ensemble at 310 K. During the equilibration stage, the Cα atoms were kept fixed. Simulations were then performed in the NPT ensemble (*P* = 1 bar, *T* = 310 K), with all atoms free to move. Atomic coordinates were saved every 10 ps. For each trajectory, 200 ns of production and the trajectories were triplicated for a cumulative trajectory duration of 3 μs.

## 4. Conclusions

In this study, we have reported the crystal structure of a hexavariant of the domain b1 of human NRP1 in complex with the KDKPPR peptide. The mutant was designed to modify the monomer assembly observed in the crystal packing of the unbound form [9,19], in which the VEGF-binding pocket of NRP1-b1 is inaccessible. Molecular dynamic trajectories permitted investigating the differences in the structures of the wild type and variant. Both structures produced similar internal flexibility and protein/peptide interaction. This showed that the ability of the protein to bind small ligands was not affected by the designed mutations. As part of our future search for ligands, we need to check that the dissociation constant (*K_D_*) of the molecules tested is the same for mutated and wild-type NRP1-b1. 

The NRP1-b1 hexavariant crystallized in a new crystal form, in which the KDKPPR peptide was not involved in the cohesion of the solid state. In the crystal, the peptide-binding site was observed to communicate with a solvent cavity large enough to diffuse small molecules. Therefore, ligand soaking in the crystal of the unbound form of NRP1-b1 hexavariant could be considered as a strategy to prepare NRP1-b1 complexes with peptides that target the pocket where VEGF binds. 

The structure of the NRP1-b1 hexavariant in complex with the KDKPPR peptide was analyzed with two original tools. First, the Nucleophilic Influence Zones (NIZ) of the ligand-binding cleft were analyzed. They revealed two additional residues (Tyr297 and Glu348) as probable attractors of the ligand electrophilic groups and two residues (Asp320 and Glu348) in the electrostatic steering of the ligand in its binding site. Secondly, the enrichment of contacts was calculated to analyze the interactions between the protein and the peptide. This metric has provided valuable insights into the diversity and specificity of the protein/ligand interaction. The complex was mainly stabilized by a notable presence of strong N-H…O and O-H…O hydrogen bonds, which were crucial due to the loop-rich nature of the VEGF-binding site. Indeed, these loops exhibited mobility both in the unbound and bound forms, as suggested by the MD simulation.

## Figures and Tables

**Table 1:** Statistical analysis of intermolecular contacts on the Hirshfeld interface between the PPR moiety of the KDKPPR peptide and NRP1-b1.

Atom Type	C	Hc	N	Ho/n	O	W ^#^
surface_peptide	8.8	**42.7**	5.5	**22.9**	**20.1**	0
surface_protein	**21.4**	**30.0**	1.2	**15.6**	**15.9**	**16.0**
C	1.4	**21.3**	1.3	3.4	0.4	1.0
Hc		**10.3**	3.5	7.2	**15.3**	4.8
N			0	0.5	0.2	1.3
Ho/n	*C*_XY_	(%)		1.6	**17.4**	6.9
O					0.3	2.0
C	0.75	**1.80**	1.02	0.54	0.08	0.73
Hc		0.81	1.62	0.53	**1.20**	0.70
N			0	0.40	0.17	1.44
Ho/n	*E*_XY_			0.44	**2.58**	**1.88**
O					0.09	0.64
		Hphob	Hphil	Hphob * Hphil		
surface %	peptide	57.1	43.0			
surface %	protein	52.6	47.4			
contacts	%	37.8	28.2	34.0		
enrichment		1.26	1.38	0.69		

* The Hirshfeld surface was limited to the regions where the electron density is larger than 0.0013 e/Å^3^ in order to omit the peptide surface exposed to the solvent. ^#^ Oxygen atom of water molecules. The second and third rows show the chemical content on the Hirshfeld surface. The next rows show the % *C*_XY_ of the contact types on the surface, followed by their enrichment ratios. The major surface components, the *C*_XY_ contacts and the significantly enriched contacts (*E* > 1) are highlighted in bold characters. In the lower part of the table, the atoms are grouped into hydrophobic (Hphob) and hydrophilic (Hphil) atoms.

**Table 2:** Statistics of X-ray diffraction data collection and model refinement.

Data Collection	
Diffraction source	ESRF FIP2-BM07
Wavelength (Å)	0.9795
Space group	*P*3_2_2
*a*, *b*, *c* (Å)	59.77, 59.77, 174.60
*α*, *β*, *γ* (°)	90, 90, 120
Resolution range (Å)	44.53–1.35 (1.37–1.35) ^1^
Total number of measured intensities	696,189 (16,486) ^1^
Number of unique reflections	80,230 (3895) ^1^
Average redundancy	8.7 (4.2) ^1^
Mean *I*/sig(*I*)	28.7 (1.9) ^1^
Completeness (%)	99.7 (95.7) ^1^
*R_merge_* ^2^; *R_meas_* ^3^	0.031 (0.681) ^1^; 0.033 (0.779) ^1^
*CC*_1/2_ ^4^	1.00 (0.69) ^1^
Wilson *B*-factor (Å^2^)	17.9 (Aimless)/21.14 (Buster)
**Refinement and structure**	
Resolution range (Å)	19.57–1.35 (1.36–1.35) ^1^
Number of reflections	80,203 (1605) ^1^
*R_work_*/*R_free_* ^5^	0.1942/0.2100 (0.2826/0.2760) ^1^
Correlation *Fo* − *Fc*/*Fo* − *Fc_free_*	0.966/0.963
Total number of atoms	2910
Average *B* factor (Å^2^)	25.55
**Model quality**	
RMSZ bond lengths ^6^	1.28
RMSZ bond angles ^6^	1.14
Ramachandran favored (%)	97.5
Ramachandran allowed (%)	2.4
Rotamer outliers (%)	1.8
Clash-score ^7^	10

^1^ Values in parentheses are for the highest resolution shell;  2Rmerge=∑h∑iIhi−〈Ih〉/∑h∑i〈Ih〉;  3Rmeas=∑h∑inhnh−11/2Ihi−〈Ih〉/∑h∑i〈Ih〉 (with $I_{hi}$ being the intensity of an individual observation of the reflection **h** and $\langle I_{h}\rangle$ being the average of all symmetry-related or replicate observations); ^4^ CC_1/2_ is the correlation coefficient of the mean intensities between two random half-sets of data. R5 work=∑hFo−Fc/∑hFo, 95% of the reflections, *R_free_* same formula (5% of the reflections) ($F_{o}$ and $F_{c}$ observed and calculated structure factors, respectively). ^6^ RMSZ: root mean square Z-score. ^7^ The MolProbity clash-score is the number of serious clashes per 1000 atoms.

## Data Availability

PDB data (8PFE) are made freely available by the wwPDB (https://www.wwpdb.org/ (accessed on 21 July 2023)).

## Supplementary Materials

The following supporting information can be downloaded at https://www.mdpi.com/article/10.3390/molecules28145603/s1, Figure S1. Large aqueous cavity in the crystal structure of NRP1-b1 hexavariant; Figure S2: Comparison of the packing of NRP1-b1 in crystal forms II (a) and III (b); Figure S3: Environment of the ligand in crystal forms II, III, IV and V; Figure S4: Comparison of atomic root mean square fluctuations (RMSFs) and root mean square thermal displacements (RMSTDs); Table S1: Intermolecular hydrogen bonds between NRP1-b1 chains in hexavariant crystal; Table S2: Crystal forms of NRP1-b1 fragment in the unbound form or in complex with small ligands.

## References

[1] Lu D., Shang G., He X., Bai X.C., Zhang X. (2021). Architecture of the Sema3A/PlexinA4/Neuropilin tripartite complex. Nat. Commun..

[2] Soker S., Miao H.-Q., Nomi M., Takashima S., Klagsbrun M. (2002). VEGF165 mediates formation of complexes containing VEGFR-2 and neuropilin-1 that enhance VEGF165-receptor binding. J. Cell. Biochem..

[3] Soker S., Takashima S., Miao H.Q., Neufeld G., Klagsbrun M. (1998). Neuropilin-1 Is Expressed by Endothelial and Tumor Cells as an Isoform-Specific Receptor for Vascular Endothelial Growth Factor. Cell.

[4] Chaudhary B., Khaled Y.S., Ammori B.J., Elkord E. (2014). Neuropilin 1: Function and therapeutic potential in cancer. Cancer Immunol. Immunother..

[5] Daly J.L., Simonetti B., Klein K., Chen K.-E., Williamson M.K., Antón-Plágaro C., Shoemark D.K., Simón-Gracia L., Bauer M., Hollandi R. (2020). Neuropilin-1 is a host factor for SARS-CoV-2 infection. Science.

[6] Lerouge L., Gries M., Chateau A., Daouk J., Lux F., Rocchi P., Cedervall J., Olsson A.K., Tillement O., Frochot C. (2023). Targeting Glioblastoma-Associated Macrophages for Photodynamic Therapy Using AGuIX((R))-Design Nanoparticles. Pharmaceutics.

[7] Liu S.D., Zhong L.P., He J., Zhao Y.X. (2020). Targeting neuropilin-1 interactions is a promising anti-tumor strategy. Chin. Med. J..

[8] Pellet-Many C., Frankel P., Jia H., Zachary I. (2008). Neuropilins: Structure, function and role in disease. Biochem. J..

[9] Lee C.C., Kreusch A., McMullan D., Ng K., Spraggon G. (2003). Crystal Structure of the Human Neuropilin-1 b1 Domain. Structure.

[10] Powell J., Mota F., Steadman D., Soudy C., Miyauchi J.T., Crosby S., Jarvis A., Reisinger T., Winfield N., Evans G. (2018). Small Molecule Neuropilin-1 Antagonists Combine Antiangiogenic and Antitumor Activity with Immune Modulation through Reduction of Transforming Growth Factor Beta (TGFbeta) Production in Regulatory T-Cells. J. Med. Chem..

[11] Mota F., Fotinou C., Rana R.R., Chan A.W.E., Yelland T., Arooz M.T., O’Leary A.P., Hutton J., Frankel P., Zachary I. (2018). Architecture and hydration of the arginine-binding site of neuropilin-1. FEBS J..

[12] Parker M.W., Xu P., Li X., Vander Kooi C.W. (2012). Structural basis for selective vascular endothelial growth factor-A (VEGF-A) binding to neuropilin-1. J. Biol. Chem..

[13] Jarvis A., Allerston C.K., Jia H., Herzog B., Garza-Garcia A., Winfield N., Ellard K., Aqil R., Lynch R., Chapman C. (2010). Small molecule inhibitors of the neuropilin-1 vascular endothelial growth factor A (VEGF-A) interaction. J. Med. Chem..

[14] Kamarulzaman E.E., Vanderesse R., Gazzali A.M., Barberi-Heyob M., Boura C., Frochot C., Shawkataly O., Aubry A., Wahab H.A. (2017). Molecular modelling, synthesis and biological evaluation of peptide inhibitors as anti-angiogenic agent targeting neuropilin-1 for anticancer application. J. Biomol. Struct. Dyn..

[15] Kamarulzaman E.E., Gazzali A.M., Acherar S., Frochot C., Barberi-Heyob M., Boura C., Chaimbault P., Sibille E., Wahab H.A., Vanderesse R. (2015). New Peptide-Conjugated Chlorin-Type Photosensitizer Targeting Neuropilin-1 for Anti-Vascular Targeted Photodynamic Therapy. Int. J. Mol. Sci..

[16] Bechet D., Mordon S.R., Guillemin F., Barberi-Heyob M.A. (2014). Photodynamic therapy of malignant brain tumours: A complementary approach to conventional therapies. Cancer Treat. Rev..

[17] Gries M., Thomas N., Daouk J., Rocchi P., Choulier L., Jubreaux J., Pierson J., Reinhard A., Jouan-Hureaux V., Chateau A. (2020). Multiscale Selectivity and in vivo Biodistribution of NRP-1-Targeted Theranostic AGuIX Nanoparticles for PDT of Glioblastoma. Int. J. Nanomed..

[18] Thomas E., Colombeau L., Gries M., Peterlini T., Mathieu C., Thomas N., Boura C., Frochot C., Vanderesse R., Lux F. (2017). Ultrasmall AGuIX theranostic nanoparticles for vascular-targeted interstitial photodynamic therapy of glioblastoma. Int. J. Nanomed..

[19] Richard M., Chateau A., Jelsch C., Didierjean C., Manival X., Charron C., Maigret B., Barberi-Heyob M., Chapleur Y., Boura C. (2016). Carbohydrate-based peptidomimetics targeting neuropilin-1: Synthesis, molecular docking study and in vitro biological activities. Bioorgan. Med. Chem..

[20] Jelsch C., Longhi S., Cambillau C. (1998). Packing forces in nine crystal forms of cutinase. Proteins Struct. Funct. Genet..

[21] Domagala S., Fournier B., Liebschner D., Guillot B., Jelsch C. (2012). An improved experimental databank of transferable multipolar atom models—ELMAM2. Construction details and applications. Acta Crystallogr. A.

[22] wwPDB consortium (2019). Protein Data Bank: The single global archive for 3D macromolecular structure data. Nucleic Acids Res..

[23] Mata I., Molins E., Espinosa E. (2007). Zero-Flux Surfaces of the Electrostatic Potential:  The Border of Influence Zones of Nucleophilic and Electrophilic Sites in Crystalline Environment. J. Phys. Chem. A.

[24] Vuković V., Leduc T., Jelić-Matošević Z., Didierjean C., Favier F., Guillot B., Jelsch C. (2021). A rush to explore protein–ligand electrostatic interaction energy with Charger. Acta Crystallogr. Sect. D Struct. Biol..

[25] Wade R.C., Gabdoulline R.R., Lüdemann S.K., Lounnas V. (1998). Electrostatic steering and ionic tethering in enzyme–ligand binding: Insights from simulations. Proc. Natl. Acad. Sci. USA.

[26] Hirshfeld F.L. (1977). Bonded-atom fragments for describing molecular charge densities. Theor. Chim. Acta.

[27] Guillot B., Enrique E., Huder L., Jelsch C. (2014). MoProViewer: A tool to study proteins from a charge density science perspective. Acta Crystallogr. Sect. A.

[28] Jelsch C., Ejsmont K., Huder L. (2014). The enrichment ratio of atomic contacts in crystals, an indicator derived from the Hirshfeld surface analysis. IUCrJ.

[29] Jelsch C., Bibila Mayaya Bisseyou Y. (2017). Atom interaction propensities of oxygenated chemical functions in crystal packings. IUCrJ.

[30] Sun Z., Liu Q., Qu G., Feng Y., Reetz M.T. (2019). Utility of B-Factors in Protein Science: Interpreting Rigidity, Flexibility, and Internal Motion and Engineering Thermostability. Chem. Rev..

[31] Ahlstrom L.S., Miyashita O. (2014). Packing interface energetics in different crystal forms of the λ Cro dimer. Proteins Struct. Funct. Bioinform..

[32] Ahlstrom L.S., Vorontsov I.I., Shi J., Miyashita O. (2017). Effect of the Crystal Environment on Side-Chain Conformational Dynamics in Cyanovirin-N Investigated through Crystal and Solution Molecular Dynamics Simulations. PLoS ONE.

[33] Janowski P.A., Liu C., Deckman J., Case D.A. (2016). Molecular dynamics simulation of triclinic lysozyme in a crystal lattice. Protein Sci..

[34] Kuzmanic A., Pannu N.S., Zagrovic B. (2014). X-ray refinement significantly underestimates the level of microscopic heterogeneity in biomolecular crystals. Nat. Commun..

[35] Alshawaf E., Hammad M.M., Marafie S.K., Ali H., Al-Mulla F., Abubaker J., Mohammad A. (2022). Discovery of natural products to block SARS-CoV-2 S-protein interaction with Neuropilin-1 receptor: A molecular dynamics simulation approach. Microb. Pathog..

[36] Appleton B.A., Wu P., Maloney J., Yin J., Liang W.-C., Stawicki S., Mortara K., Bowman K.K., Elliott J.M., Desmarais W. (2007). Structural studies of neuropilin/antibody complexes provide insights into semaphorin and VEGF binding. EMBO J..

[37] Janssen B.J.C., Malinauskas T., Weir G.A., Cader M.Z., Siebold C., Jones E.Y. (2012). Neuropilins lock secreted semaphorins onto plexins in a ternary signaling complex. Nat. Struct. Mol. Biol..

[38] Kabsch W. (2010). XDS. Acta Crystallogr. Sect. D Biol. Crystallogr..

[39] Evans P.R., Murshudov G.N. (2013). How good are my data and what is the resolution?. Acta Crystallogr. D Biol. Crystallogr..

[40] Winn M.D., Ballard C.C., Cowtan K.D., Dodson E.J., Emsley P., Evans P.R., Keegan R.M., Krissinel E.B., Leslie A.G.W., McCoy A. (2011). Overview of the CCP4 suite and current developments. Acta Crystallogr. Sect. D Biol. Crystallogr..

[41] Vagin A., Teplyakov A. (2010). Molecular replacement with MOLREP. Acta Crystallogr. Sect. D Biol. Crystallogr..

[42] Emsley P., Lohkamp B., Scott W.G., Cowtan K. (2010). Features and development of Coot. Acta Crystallogr. Sect. D Biol. Crystallogr..

[43] Smart O.S., Womack T.O., Flensburg C., Keller P., Paciorek W., Sharff A., Vonrhein C., Bricogne G. (2012). Exploiting structure similarity in refinement: Automated NCS and target-structure restraints in BUSTER. Acta Crystallogr. Sect. D.

[44] Williams C.J., Headd J.J., Moriarty N.W., Prisant M.G., Videau L.L., Deis L.N., Verma V., Keedy D.A., Hintze B.J., Chen V.B. (2018). MolProbity: More and better reference data for improved all-atom structure validation. Protein Sci..

[45] Laskowski R.A., Swindells M.B. (2011). LigPlot+: Multiple Ligand–Protein Interaction Diagrams for Drug Discovery. J. Chem. Inf. Model..

[46] Voss N.R., Gerstein M. (2010). 3V: Cavity, channel and cleft volume calculator and extractor. Nucleic Acids Res..

[47] Phillips J.C., Braun R., Wang W., Gumbart J., Tajkhorshid E., Villa E., Chipot C., Skeel R.D., Kalé L., Schulten K. (2005). Scalable molecular dynamics with NAMD. J. Comput. Chem..

[48] Best R.B., Zhu X., Shim J., Lopes P.E.M., Mittal J., Feig M., MacKerell A.D. (2012). Optimization of the Additive CHARMM All-Atom Protein Force Field Targeting Improved Sampling of the Backbone ϕ, ψ and Side-Chain χ1 and χ2 Dihedral Angles. J. Chem. Theory Comput..

[49] Jorgensen W.L., Chandrasekhar J., Madura J.D., Impey R.W., Klein M.L. (1983). Comparison of simple potential functions for simulating liquid water. J. Chem. Phys..

[50] Darden T., York D., Pedersen L. (1993). Particle mesh Ewald: An N⋅log(N) method for Ewald sums in large systems. J. Chem. Phys..

[51] Ryckaert J.-P., Ciccotti G., Berendsen H.J.C. (1977). Numerical integration of the cartesian equations of motion of a system with constraints: Molecular dynamics of n-alkanes. J. Comput. Phys..

