# Supplementary material for: New Crystal Form of Human Neuropilin-1 b1 Fragment with Six Electrostatic Mutations Complexed with KDKPPR Peptide Ligand
Source: Molecules. 2023 Jul 24;28(14):5603. doi: 10.3390/molecules28145603 (PMC10385628; doi:10.3390/molecules28145603)

Supplementary Materials

New crystal form of Human Neuropilin-1 b1 fragment with six electrostatic mutations complexed with KDKPPR peptide ligand

Goudiaby et al.

Table of contents

Table S1. Intermolecular hydrogen bonds between NRP1-b1 chains in hexavariant crystal... 2

Table S2. Crystal forms of NRP1-b1 fragment in the unbound form or in complex with small ligands 3

Figure S1. Large aqueous cavity in the crystal structure of NRP1-b1 hexavariant. 4

Figure S2. Comparison of the packing of NRP1-b1 in crystal forms II (a) and III (b). 5

Figure S3. Environment of the ligand in crystal forms II, III, IV and V. 6

Figure S4. Comparison of atomic root mean square fluctuations (RMSF) and root mean square thermal displacements (RMSTD) 7

Table S1. Intermolecular hydrogen bonds between NRP1-b1 chains in hexavariant crystal.

Residue 1	Residue 2	Distance (Å)
NZ-K277(A)	OXT-T427(A)[i]	2.67
O-E282(A)	OH-Y322(A)[ii]	2.56
O-S283(A)	OG-S321(A)[ii]	2.78
NZ-K285(A)	OE1-E319(A)[ii]	2.63
NH1-R323(A)	OD1-D389(C)[iii]	2.85
NH2-R323(A)	OD2-D389(C)[iii]	2.85
NZ-K352(A)	O-S294(C)[iv]	3.05
OD1-D389(A)	NH1-R323(C)[iv]	2.77
OD2-D389(A)	NH2-R323(C)[iv]	2.70
N-K425(A)	O-T427(A)[i]	2.76
O-K425(A)	N-T427(A)[i]	2.90
O-E282(C)	OH-Y322(C)[v]	2.58
O-S283(C)	OG-S321(C)[v]	2.99

Symmetry Codes : (i) -1+Y, 1+X, -Z (ii) -1+Y, X, -Z (iii) Y, 1+X, -Z (iv) 1+X-Y, 1-Y, -Z+1/3 ; (v) 1+X-Y, 1-Y, -Z+1/3

Table S2. Crystal forms of NRP1-b1 fragment in the unbound form or in complex with small ligands

Form	Z'	Space group	a, b, c (Å), α, β, γ (°)	Res. (Å)*	PDB entries
I	1	$P4_12_12$	62.3, 62.3, 86.0, 90, 90, 90	1.45	**5C7G**[1], **4RN5**[2], **1KEX**[3],
II[#]	2	$P2_1$	40.7, 89.2, 41.6, 90, 99, 90	1.38	**5JGI**[4], **5J1X**[5$], **5IJR**[6], **5JHK**[7], **5JGQ**[8], **5IYY**[9], **3I97**[10]
III[#]	1	$P4_1$	43.4, 43.4, 91.2, 90, 90, 90	0.9	**6FMC**[11#]
IV	1	$P2_12_12_1$	38.9, 40.0, 97.6, 90, 90, 90	1.06	**6TKK**[12]
V	4	$P2_12_12$	89.9, 89.9, 108.3, 90, 90, 90	2.36	**7JJC**[13]
VI	2	$P3_221$	59.8, 59.8, 174.6, 90, 90, 120	1.35	**8PFE**[14]

* Highest resolution obtained in a crystal form; $ the crystal form of 5J1X is slightly different from that of form II. The *c* parameter has doubled, the space group remains $P2_1$ and Z′ is equal to 4; # The packing of NRP1-b1 domain is identical in crystal forms II and III (see Figure S2).

Ligands observed in the VEGF pocket:

[1] Bicine; [2] Acetate ion; [3] No ligand; [4] *N*-alpha-l-acetyl-arginine, isomeric SMILES: (CC(=O)N[C@@H](CCCNC(=N)N)C(=O)O); [5] *N*-2-(*tert*-butoxycarbonyl)-L-arginine, isomeric SMILES: ([H]/N=C(/N)\NCCC[C@@H](C(=O)O)NC(=O)OC(C)(C)C); [6] L-homoarginine, isomeric SMILES: (C(CCNC(=N)N)C[C@@H](C(=O)O)N); [7] *N*-(benzenecarbonyl)glycyl-L-arginine, isomeric SMILES: [H]/N=C(/N)\NCCC[C@@H](C(=O)O)NC(=O)CNC(=O)c1ccccc1; [8] *N*~2~-(benzenecarbonyl)-L-arginine, isomeric SMILES: [H]/N=C(/N)\NCCC[C@@H](C(=O)O)NC(=O)c1ccccc1; [9] 7 *N*~2~-[(benzyloxy)carbonyl]-L-arginine, isomeric SMILES: [H]/N=C(\N)/NCCC[C@@H](C(=O)O)NC(=O)OCc1ccccc1; [10] (*S*)-2-(3-(benzo[c][1,2,5]thiadiazole-4-sulfonamido)thiophene-2-carboxamido)-5-guanidinopentanoic acid, isomeric SMILES [H]/N=C(/N)\NCCC[C@@H](C(=O)O)NC(=O)c1c(ccs1)NS(=O)(=O)c2cccc3c2nsn3; [11] (2~{*S*})-2-[[3-[[5-[4-(aminomethyl)phenyl]-1-benzofuran-7-yl]sulfonylamino]thiophen-2-yl]carbonylamino]-5-carbamimidamido-pentanoic acid, isomeric SMILES: [H]/N=C(/N)\NCCC[C@@H](C(=O)O)NC(=O)c1c(ccs1)NS(=O)(=O)c2cc(cc3c2occ3)c4ccc(cc4)CN; **12** Ace-Arg-Pro-Gln-Pro-Arg; **13** Asn-Ser-Pro-Arg-Arg-Ala-Arg; **14** Lys-Asp-Lys-Pro-Pro-Arg.

Figure S1. Large aqueous cavity in the crystal structure of NRP1-b1 hexavariant.
Top: Stereoview of a large aqueous cavity. A cavity contains two peptides. The electron density map indicates that the KDK moiety of the peptide is not highly ordered within this cavity. The NRP1-b1 chains are shown as a surface and the PPR moiety of the peptides is depicted as sticks.

Bottom: Stereo-view that highlights the volume of the cavity. A volume of approximatively 30,000 Å^3 was estimated using 3V (Voss and Gerstein, 2010, NAR, DOI 10.1093/nar/gkq395)

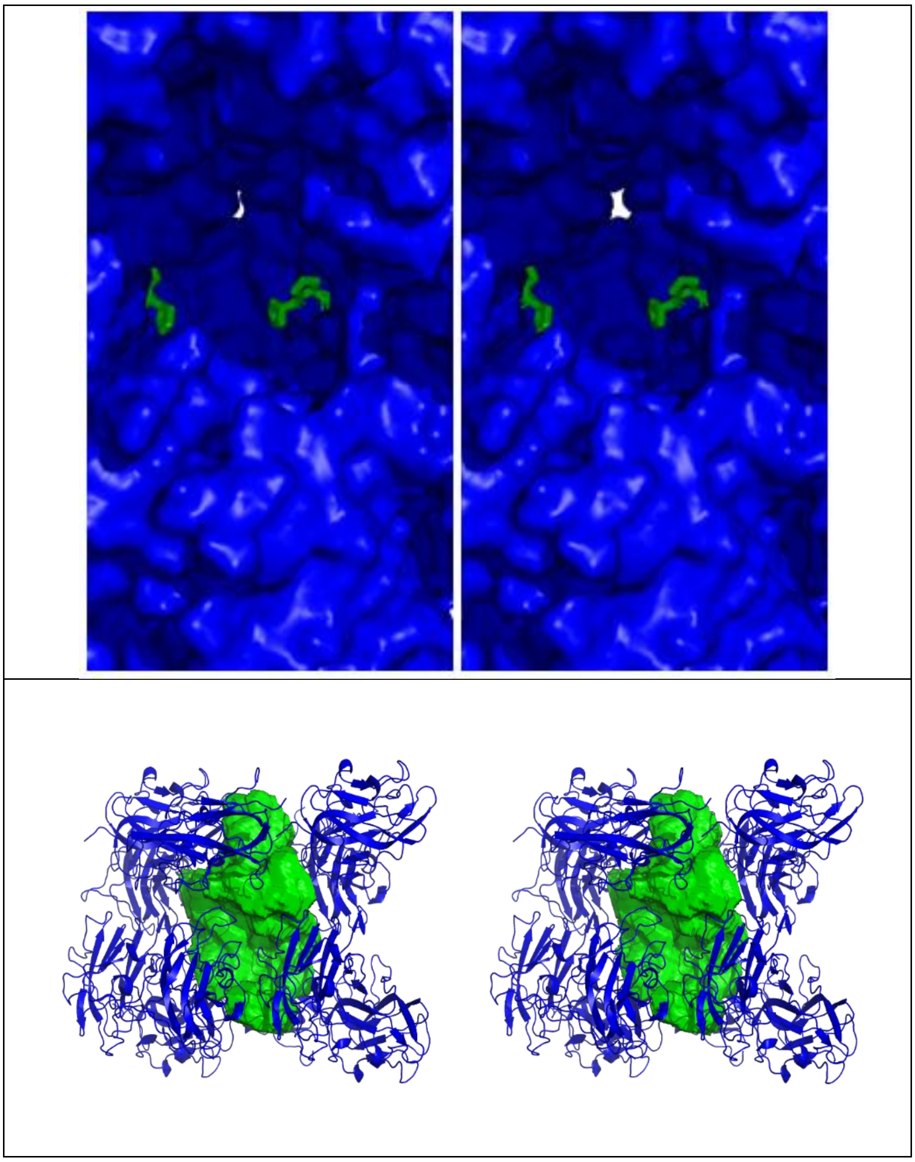

Figure S2. Comparison of the packing of NRP1-b1 in crystal forms II (a) and III (b).
The figure shows that both crystal forms have the same molecular packing pattern. NRP1-b1 chains are presented in ribbon mode. The left and right parts were constructed using PDB entries 5JGI and 6FMC, respectively.

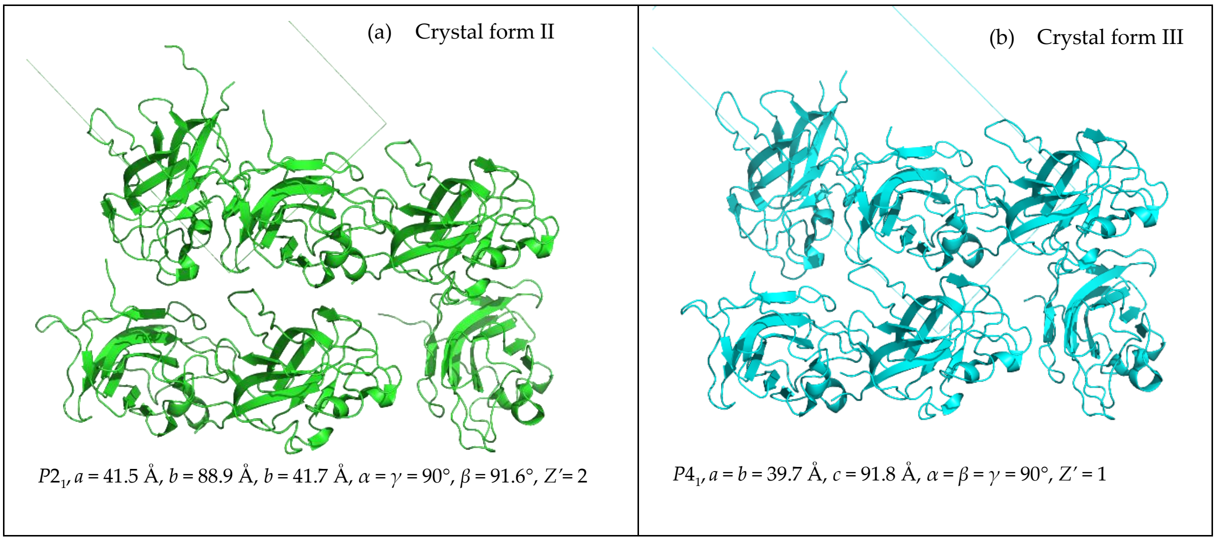

Figure S3. Environment of the ligand in crystal forms II, III, IV and V.

The figures depict ligands within the VEGF-binding pocket. In all cases, at least one ligand is involved in maintaining the cohesion of the crystal packing. The bonds of the ligands are colored in blue. The labels of the symmetry-related molecules are colored in green and displayed in bold characters. Hydrogen bonds are illustrated as dashed lines. The figures were generated using Ligplot+ (Laskowski & Mark, 2011, J. Chem. Inf. Model., DOI 10.1021/ci200227u).

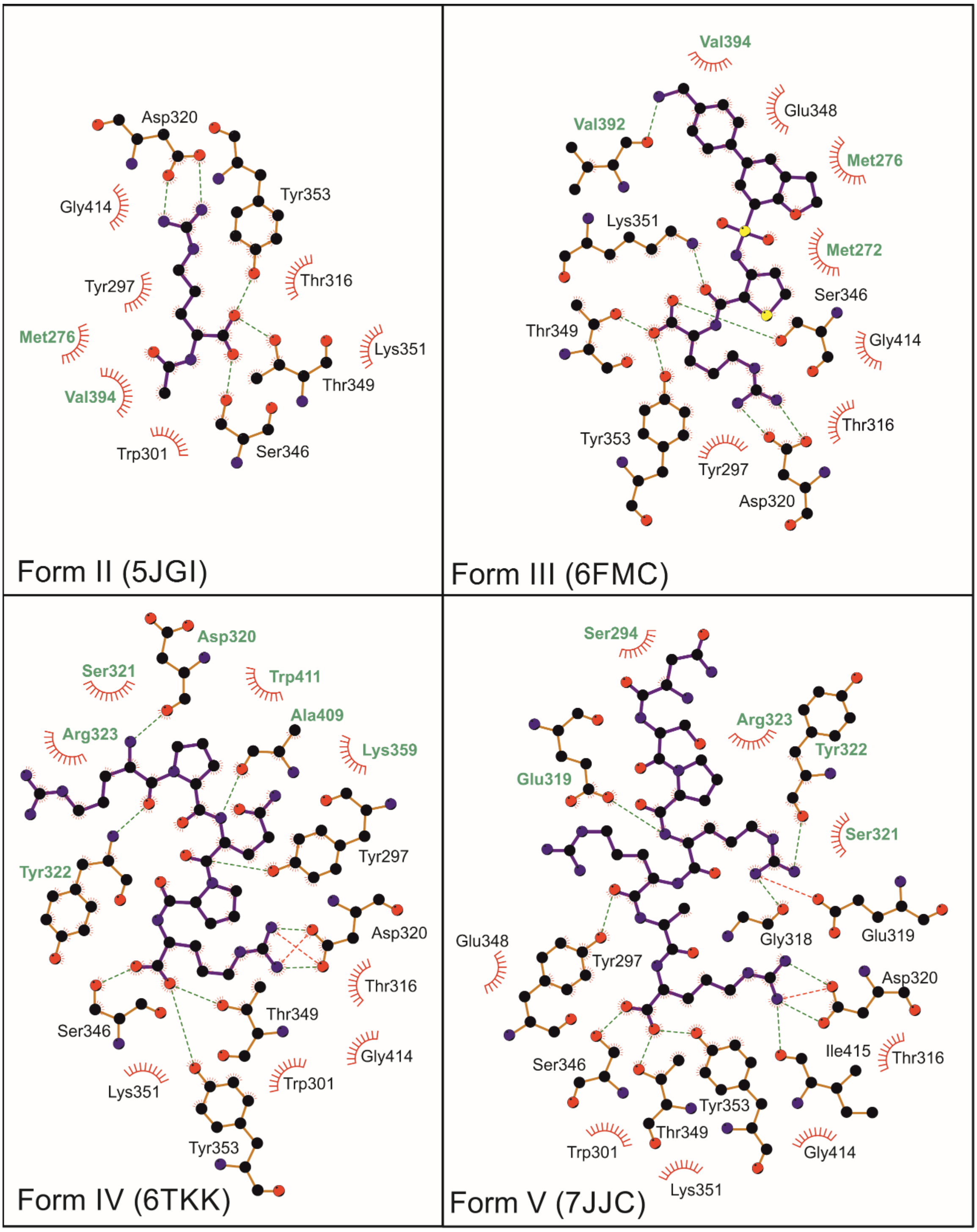

Figure S4. Comparison of atomic root mean square fluctuations (RMSF) and root mean square thermal displacements (RMSTD)

RMSF and RMSD of the Cα atom positions averaged, respectively, over the 3 molecular dynamics simulations of NRP1 (holo, 6mut) and over the 2 monomers of the crystal structure. The RMSTD values are derived from the thermal parameters *B*. (a) values as a function of residue numbers, (b) scatterplot of (RMSF,RMSD).

(a)

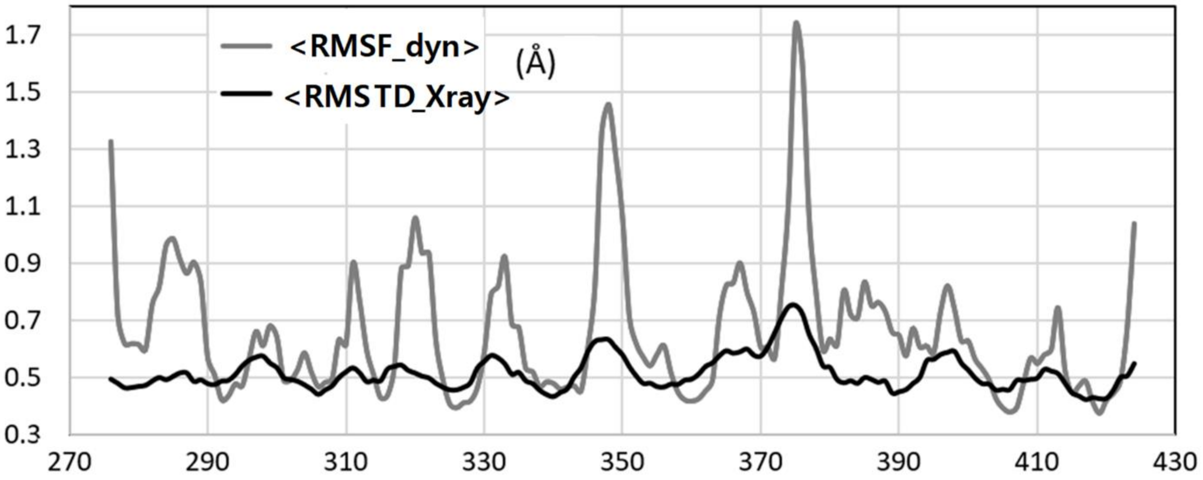

(b)

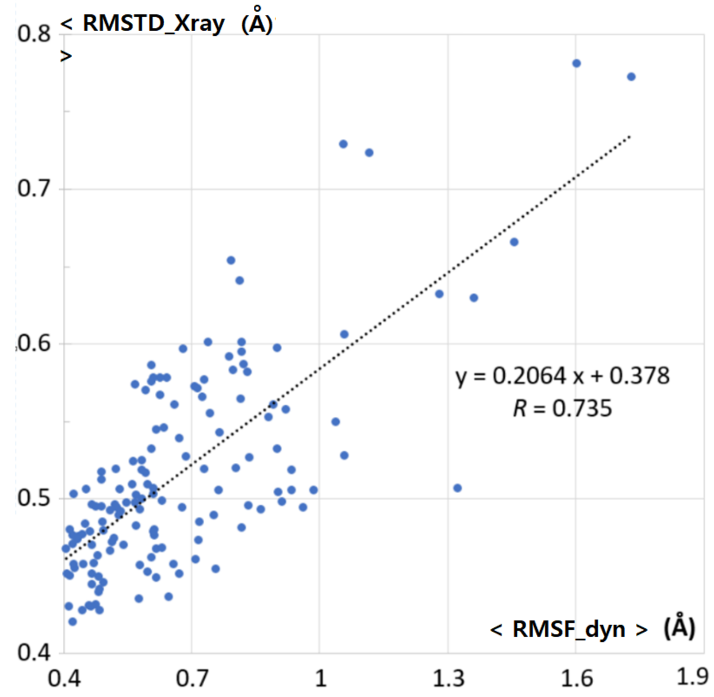

Supplement: Supplementary file 1 [file molecules-28-05603-s001.zip › molecules-2493973-supplementary.pdf]
